# Dietary Supplement for Core Symptoms of Autism Spectrum Disorder: Where Are We Now and Where Should We Go?

**DOI:** 10.3389/fpsyt.2017.00155

**Published:** 2017-08-23

**Authors:** Yong-Jiang Li, Jian-Jun Ou, Ya-Min Li, Da-Xiong Xiang

**Affiliations:** ^1^Department of Pharmacy, The Second Xiangya Hospital, Central South University, Changsha, China; ^2^Institute of Mental Health, The Second Xiangya Hospital, Central South University, Changsha, China; ^3^Clinical Nursing Teaching and Research Section, The Second Xiangya Hospital, Central South University, Changsha, China

**Keywords:** autism spectrum disorder, behavioral symptoms, dietary supplements, intervention, nutrition

## Abstract

Autism spectrum disorders (ASDs) are a class of severe and chronic conditions and core symptoms are deficits in social interaction, language communication impairments, and repetitive/stereotyped behavior. Given the limitations of available treatments and substantially increased prevalence of the disease, additional interventions are needed. Since the use of dietary supplements for ASD is of high prevalence, up-to-date information about those supplements are required for both parents and clinicians. Relevant articles were identified through a systematic search of PubMed, EMBASE, Cochrane library, and PsychINFO databases (through May 2017). Current best evidences of 22 randomized controlled trials on 8 different dietary supplements for core symptoms of ASD were reviewed. For each supplement, this report focuses on the definition and potential therapeutic mechanisms, the latest advances, and discussion of study limitations and future directions. Most studies were small and short term, and there is little evidence to support effectiveness of dietary supplements for children with ASD.

## Introduction

Autism spectrum disorder (ASD) is defined as a neurodevelopmental condition characterized by social communication impairment, delayed development, social function deficit, and repetitive behavior (1). The substantially increased prevalence of ASD (2, 3) and the associated heavy economic burden (4) provide a strong rationale for developing effective treatment strategies of core symptoms of ASD. To date, no medication is currently available for the core symptoms, and there is an urgent public health need for additional interventions (5, 6). While the exact etiology of ASD remains unknown, genetic, neurologic, metabolic, and immunologic factors are involved in the complex pathogenesis (7, 8). Besides, studies have observed several nutritional deficient such as vitamin D (9) and omega-3 fatty acids (10) that may occur in children with ASD. This provides a novel strategy (dietary supplements) as a complementary and alternative treatment for ASD.

Feeding problems, such as food selectivity and unusual eating patterns, are of high prevalence in children with ASD (11). Food selectivity has become a concern for its harmful influence in nutrient adequacy and inadequate nutrient intake has been reported to be associated with food selectivity in children with ASD (12). Food selectivity may further result in nutritional deficiency and the impact could be severe (13). Therefore, dietary supplements are widely used to complement nutritional deficits in children with ASD. Also, considering the early onset and chronic nature of ASD (14), dietary supplements might be the priority selection for families (15), as it can be early or long-time administrated in younger children, and it is also relatively safe, cheap, effective, and time saving (16). However, family members and caregivers are often hard to have a clear understanding of the safety and potential benefit of those supplements.

During the past years, an increasing number of studies have been conducted for searching and testing novel supplements for ASD and have yielded inconsistent results. But the use of dietary supplements intervention for ASD is of high prevalence (17). Therefore, up-to-date information about those supplements that are being studied are required for both parents and clinicians.

This paper will focus on recent insights into the treatment effects of dietary supplements on core symptoms of ASD (Table 1). For each supplement, this report will summarize the definition and potential therapeutic mechanisms, the latest advances, and discussion of study limitations and future directions. Besides, only findings from randomized controlled trials (RCTs) with rigorous methodology that have used a blind method with appropriate control groups will be discussed, open-label studies were not included in case of significant placebo effect of dietary supplement (18).

**Table 1:** Summary of published randomized controlled trials of dietary supplement interventions for ASD.

Supplement	Reference	Study design	Subjects (*N*, male percentage, age range in years)	Intervention doses and duration	Behavior outcome measure	Primary results	Serious adverse effects
Vitamin B12	Bertoglio et al. (34)	Double-blind, placebo-controlled, crossover	30 (93%), 3–8	64.5 µg/kg injection, every third day, crossover 6 weeks	PIA-CV, CGI-I, CARS, PPVT-III, Stanford Binet Fifth Edition Routing Subsets, ABC, CBCL, and MCDI	Better CGI-I score, no significant improvement in other measures	None
	Hendren et al. (23)	Double-blind, placebo-controlled	57 (79%), 3–7	75 µg/kg injection, every third day, 8 weeks	CGI-I, ABC, SRS	Better CGI-I score, no improvement in ABC and SRS	None

Vitamin D3	Saad et al. (48, 136)	Double-blind, placebo-controlled	109 (78%), 3–10	300 IU vitamin D3/kg/day, 4 months	ABC, CARS, ATEC, SRS	Significant improvements in all measures	None

Omega-3 fatty acid	Amminger et al. (101)	Double-blind, placebo-controlled	13 (100%), 5–17	840 mg/day EPA + 700 mg/day DHA, 6 weeks	ABC	No significant improvement in ABC	None
	Yui et al. (105)	Double-blind, placebo-controlled	13 (92%), 6–28	120 or 240 mg/day ARA + 120 or 240 mg/day DHA, 16 weeks	ABC, ADI-R, SRS	Significant improvement in all measures	None
	Bent et al. (103)	Double-blind, placebo-controlled	27 (89%), 3–8	0.7 g/day EPA + 0.46 g/day DHA, 12 weeks	ABC PPVT EVT BASC, SRS CGI-S	No significant differences in all measures	None
	Bent et al. (104)	Double-blind, placebo-controlled	57 (88%), 5–8	0.7 g/day EPA + 0.46 g/day DHA, 12 weeks	ABC, SRS, CGI-I	Significant improvement in stereotypy and lethargy subscales of ABC, no significant differences in other measures	None
	Voigt et al. (106)	Double-blind, placebo-controlled	48 (83%), 3–10	200 mg/day DHA, 6 months	CGI-I, ABC, CDI, BASC	No significant improvement in CGI-I across groups and in all other measures across groups	None
	Mankad et al. (102)	Double-blind, placebo-controlled	37 (73%), 2–5	1.5 g/day EPA + DHA, 6 months	PDDBI, BASC-2, CGI-I, VABS-II, PLS-4	No significant improvement in all measures	None

Probiotic and digestive enzyme	Munasinghe et al. (119)	Double-blind, placebo-controlled, crossover	43 (84%), 3–8	Proteolytic enzymes: two caps per meal, max 9 caps, crossover 3 months	GBRS	No significant improvement in all measures	None
	Parracho et al. (118)	Double-blind, placebo-controlled, crossover	22 (91%), 4–16	Probiotics: 4.5 × 10^10^ CFU/day, crossover 6 weeks	DBC, TBPS	Significant improvement in TBPS, no improvement in DBC	None
	Saad et al. (120)	Double-blind, placebo-controlled	101(81%), 3–9	Digestive enzyme, 3 months	CARS, GBRS	Significant improvement in in emotional response, general impression autistic score of CARS, and in general behavior and gastrointestinal symptoms of GBRS	None

Folinic acid	Frye et al. (55, 58)	Double-blind, placebo-controlled	48 (82%), mean 7.3	Folinic acid: 2 mg/kg/day, max 50 mg, 12 weeks	CELF-preschool-2, CELF-4, PLS-5, OACIS, VABS, ABC, SRS, BASC, AIM, ASQ	Significant improvement in verbal communication, VABS, ABC, ASQ, BASC, and better for FRAA positive participants	None

Camel milk	Bashir and Al-Ayadhi (66); AL-Ayadhi and Elamin (64)	Double-blind, placebo-controlled	45 (89%), 2–12	500 ml/day, 2 weeks	CARS	Significant improvement in CARS in raw camel milk group	None

Sulforaphane	Singh et al. (131)	Double-blind, placebo-controlled	29 (100%), 13–27	50–150 μmol/day, 18 weeks	ABC, SRS, CGI-I, OACIS	Significant improvement in all measures	None

GFCF diet	Elder et al. (77)	Double-blind, placebo-controlled, crossover	15 (80%), 2–16	GFCF diet, crossover 6 weeks	CARS, ECO	No significant improvement in all measures	None
	Whiteley et al. (74)	Single-blind	72 (89%), 4–10	GFCF diet, 12 months	ADOS, GARS, VABS, ADHD-IV	No significant improvement in all measures	None
	Knivsberg et al. (73)	Single-blind	20, mean 7.4	GFCF diet, 12 months	DIPAB	Significant improvement in DIPAB	Not reported
	Johnson et al. (75)	Single-blind, placebo-controlled	22 (82%), 3–5	GFCF diet, 3 months	Mullen scales of early learning AGS edition, CBCL, direct behavior observation measure	No significant improvement in all measures	None
	Hyman et al. (76)	Double-blind, placebo-controlled	14 (86%), 3–5	GFCF diet, 12 weeks	RFRLRS, Conner’s Abbreviated Rating Scale	No significant improvement in all measures	None

Gluten and casein supplementation	Pusponegoro et al. (78)	Double-blind, placebo-controlled	50 (88%), 3–7	11 g of gluten and 12 g of casein, 1 weeks	AWPC, PDDBI	No significant improvement in all measures	None

*ABC, aberrant behavior checklist; ADI-R, Autism Diagnostic Interview-Revised; ADOS, Autism Diagnostic Observation Scale; AWPC, approach withdrawal problems composite; ADHD-IV, Attention-Deficit Hyperactivity Disorder-IV Scale; AIM, autism impact measure; ASQ, Autism Symptoms Questionnaire; ATEC, Autism Treatment Evaluation Checklist; BASC, Behavior Assessment Scale for Children; CBCL, Child Behavior Checklist; CDI, Child Development Inventory; CARS, Childhood Autism Rating Scale; CELF, Clinical Evaluation of Language Fundamentals; CGI-I, Clinical Global Impression Scale of Improvement; DBC, Development Behavior Checklist; DIPAB, Diagnose of Psykotisk Adfaerd hosBørn (Diagnosis of Psychotic Behavior in Children); ECO, Ecological Communication Orientation; EVT, Expressive Vocabulary Test; FRAA, folate receptor-α autoantibody; GARS, Gilliam Autism Rating Scale; GBRS, Global Behavior Rating Scale; MCDI, MacArthur Communication Developmental Inventory; OACIS, Ohio Autism Clinical Impression Scale; PIA-CV, Parent Interview for Autism-Clinical Version; PPVT, Peabody Picture Vocabulary Test; PPVT-III, Peabody Picture Vocabulary Test-Third Edition; PDDBI, Pervasive Developmental Disorders Behavioral Inventory; PLS, Preschool Language Scale; RFRLRS, Ritvo-Freeman Real Life Rating Scales; SRS, Social Responsiveness Scale; TBPS, Total Behavior Problem Score; VABS, Vineland Adaptive Behavior Scale; GFCF, gluten-free and casein-free; ARA, arachidonic acid; DHA, docosahexaenoic acid; EPA, eicosapentaenoic acid*.

## Methods

**Figure 1:**
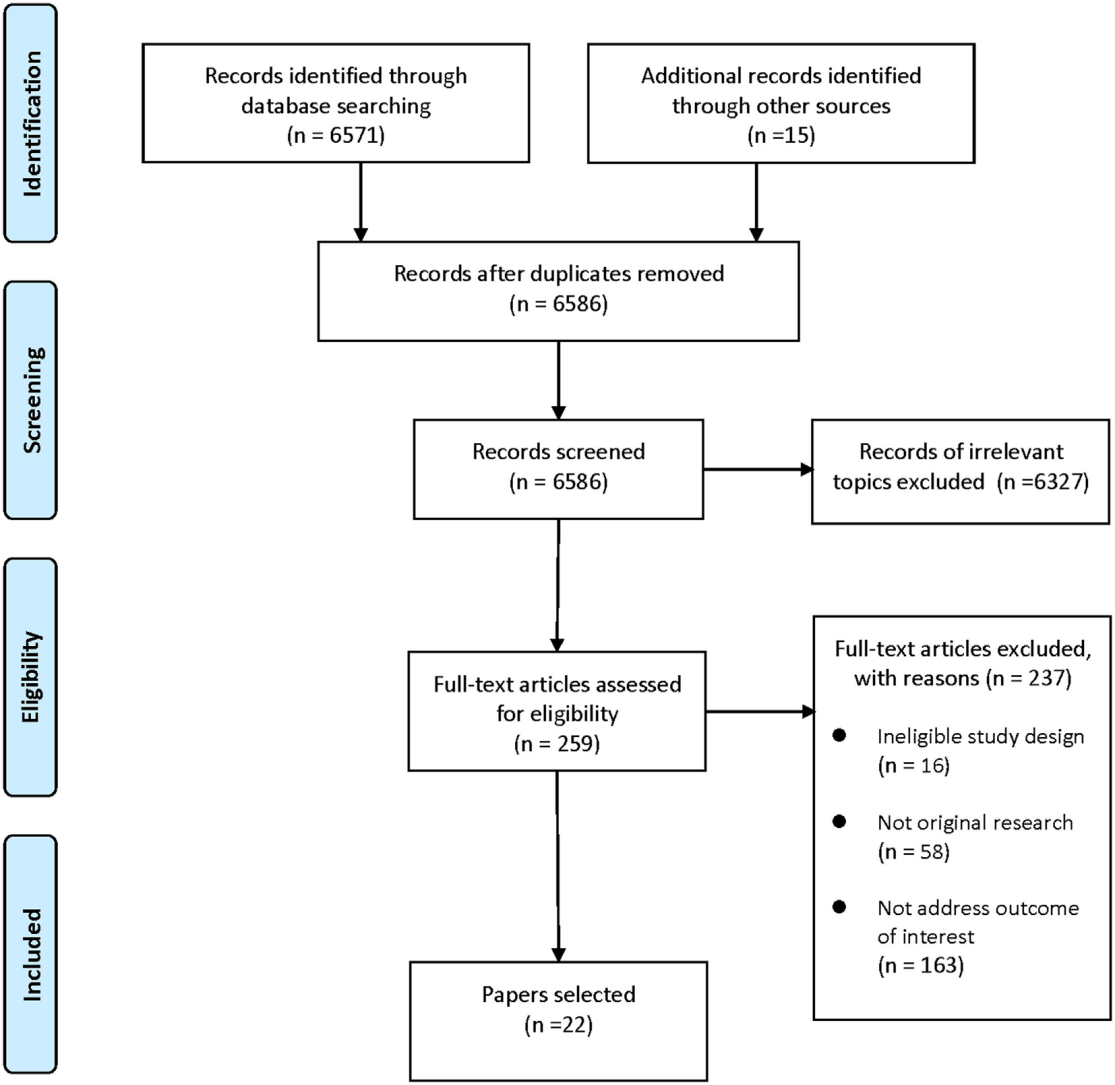
Flow diagram of the study selection process.

A systematic search of PubMed, EMBASE, Cochrane library, and PsycINFO databases was conducted to retrieve studies on dietary supplement interventions for ASD published in the last two decades (through May 2017). The following terms were used: “autism/Autism Spectrum Disorder/autistic/ASD” and “intervention/therapy/treatment/supplement/dietary.” Reference lists of selected articles and relevant reviews were also hand searched. Articles were screened and selected according to the following eligibility criteria: (1) the study was a RCT; (2) the study addressed dietary supplements intervention for ASD; (3) outcome of interest was core symptoms of ASD. Our first search provided 6,571 records. After excluding duplicates and records of irrelevant topics, 259 articles remained for full-text review. Papers without original data (e.g., reviews and meta-analyses) and studies not RCTs or not addressed outcome of interest were also excluded. Thus, this filtered search identified 22 articles. The study selection process was presented in Figure 1.

## Results

### Methyl B12

Methyl B12 is a vital cofactor for the regeneration of methionine from homocysteine by providing methyl groups for the transmethylation and transsulfuration metabolic pathways (19, 20). Deficiency of methyl B12 may lead to increased levels of homocysteine and decreased levels of methionine and *S*-adenosylmethionine (SAM) (21–23). SAM deficiency could also restrict the transsulfuration pathway and thus aggravate the accumulation of homocysteine. Furthermore, cysteine and GSH are products of the transsulfuration pathway (22, 23), reduced synthesis of them will consequently decrease antioxidant capacity as GSH is a key antioxidant (24, 25).

Studies have shown specific deficits in metabolic processes in children with ASD, including both cellular methylation (26) and glutathione-mediated antioxidant defense (27, 28). Laboratory changes, including lower plasma methionine, SAM, homocysteine, cystathionine, cysteine, and total glutathione, were also noted in children with ASD (26, 29). Abnormal transsulfuration metabolism and reduced antioxidant capacity were also noted in ASD (30). Therefore, vitamin B12 has been used for elevating methylation capacity and improving the “redox status” of children with ASD. Reversal of those biochemical abnormities by methyl B12 supplementation may improve clinical behavioral outcomes.

Furthermore, vitamin B12 plays as a significant role in inhibiting air pollutant, nitrous oxide (N_2_O), which was reported as a risk factor for ASD (31). Gestational exposure to N_2_O activates certain opioid receptor subtypes and induces physiological actions that were associated with many known autistic symptoms (31, 32). Moreover, N_2_O could oxidize the cobalt ion irreversibly within cobalamin and leave vitamin B12 inactive (33). Therefore, supplementation of vitamin B12 might be of help for the reduced vitamin B12 level and elevated homocysteine levels and other metabolic processes in ASD induced by N_2_O exposure.

Hendren et al. have recently published their findings from a double-blind placebo-controlled trial on 57 children aged 3–7 years with ASD (23). The results showed that methyl B12 supplementation improved symptoms of ASD as the clinician rated Clinical Global Impression Scale of Improvement (CGI-I) score was statistically significantly better (lower) in the methyl B12 group (mean: 2.4) than in the placebo group (mean: 3.1), while no improvement was observed in the parent-rated aberrant behavior checklist (ABC) or Social Responsiveness Scale (SRS). What’s more, the better CGI-I score was positively correlated with increased levels of methionine, decreased *S*-adenosyl-lhomocysteine (SAH) in plasma, and a better ratio of SAM to SAH, which indicated an improvement in cellular methylation capacity. Those findings were consistent with what reported in their prior published paper of a pilot study using a crossover design (34). Limitations of the two studies were small sample sizes and inadequate laboratory testing.

Methyl B12 supplementation appears to be safe and might be helpful for some symptoms of ASD; however, delivery of methyl B12 in the two trials were subcutaneous injection in every 3 days, and there is no study of oral or nasal administration of methyl B12 on autistic patient. Although subcutaneous injectable methyl B12 appears to be safe and effective, it is also invasive and may be with poor compliance. Oral taken of methyl B12 is considered to be more suitable for dietary supplementation (35), but thought to be less effective because oral absorption does not ensure consistent high concentrations. Further study is required to determine whether methyl B12 could be used as a dietary supplement for ASD.

### Vitamin D

Vitamin D is a kind of steroidal derivatives and is also a neuroactive steroid that involves in the development of brain (36), having multi biological activities, including cellular proliferation, differentiation, calcium signaling, neurotrophic, and neuroprotective actions (9); it also plays an essential role in myelination (37) and thus appears to have effects on neurotransmission and synaptic plasticity (9, 38).

Mounting numbers of studies have reported associations of ASD with decreased vitamin D levels in patients (39, 40), with decreased maternal vitamin D levels during pregnancy (41, 42) and even with decreased solar ultraviolet exposure (43). Possible mechanisms for vitamin D that helping prevent and treat ASD have been recently comprehensively reviewed (9, 44, 45). Two explanations are the most proposed. The first is the anti-inflammatory effects of vitamin D in the brain (46), and the second is its effects on serotonin (downregulate peripheral tryptophan hydroxylase 1 and upregulate central tryptophan hydroxylase 2) (47).

Although the association between ASD and vitamin D has been extensively studied, only one RCT has been published (48). This trial included 109 children aged 3–10 years with ASD. The dose of vitamin D was 300 IU/kg/day, not exceed 5,000 IU/day. After 4 months of supplementation, mean 25(OH)D in the treatment group was significantly increased, but not in the placebo group. As for behavioral measures, rated scores of ABC, CARS, Autism Treatment Evaluation Checklist, and SRS were all significantly better in children supplemented with vitamin D.

Findings from the only RCT suggested that oral vitamin D supplementation could safely improve 25(OH)D levels as well as improve core symptoms of ASD. While the data are preliminary, it may be recommended for children with ASD with a dose that works in the trial, especially for autistic patients with vitamin D deficiency.

### Folinic Acid

Folic acid, an essential water-soluble B vitamin, is closely related to the metabolism of homocysteine and glutathione (48, 49). As discussed earlier, homocysteine and glutathione abnormalities are associated with ASD. Thus, disruptions in folate-related metabolism may increase the risk of ASD. Several abnormalities in the metabolism of folic acid have been linked to ASD (50), and a recent review has evidenced the association between maternal supplementation with folic acid during the pregnancy and decreased risk of ASD in offspring (51).

Folinic acid is a reduced form of folate, intake of folinic acid could normalize folate-dependent one-carbon metabolism that may help stabilize cerebrospinal fluid (CSF) folate concentrations and significantly improve neurological symptoms (52–54). Folinic acid can readily enter the folate cycle without reduction (54). The folate receptor-α (FRα), which is responsible for transportation of folic acid into the brain, might be blocked in folate receptor-α autoantibody (FRAA)-positive patients or due to mitochondrial dysfunction or genetic mutations (55, 56), and thus decrease CSF folate concentrations. However, folinic acid is able to cross the blood–brain barrier through reduced folate carrier, though high-serum concentrations are required because of lower affinity than FRα (57).

There is no published RCT that has evaluated the effects of folic acid intervention on the treatment of autistic children. However, one recent randomized double-blind placebo-controlled trial has tested high-dose folinic acid for 12 weeks and found significant improvements in verbal communication (58). In that study, 48 children with ASD and language impairment were randomized to receive folinic acid (2 mg/kg/day, maximum 50 mg/day; *n* = 23) or placebo (*n* = 25). FRAA status of the children was subtyped. The investigators used various outcome measures of verbal communication (CELF, PLS) and behavioral assessment (Ohio Autism Clinical Impression Scale, VABS, ABC, SRS, Behavior Assessment Scale for Children, Autism Impact Measure, and Autism Symptoms Questionnaire) and found significant improvements in verbal communication, and core symptoms of ASD in those receiving folinic acid when compared with those receiving placebo; what’s more, the improvements were significantly greater for FRAA-positive participants.

Although folinic acid was well tolerated with very few adverse effects, the sample size was small and treatment duration was not long enough. Since folinic acid may become increasingly used to treat ASD in the future, its safety should be further ensured. Besides, clinicians should be cautious about the interaction of folinic acid and methyl B12 as they are metabolically related (19, 22, 59).

### Camel Milk

Camel milk, an important source of nutrient in some countries, has a unique composition that differs from other milk. Compared to cow milk, it does not contain beta-casein and beta-lactoglobulin, two powerful allergens (60), and thus makes it a good choice for children suffer from milk allergies (61, 62). Furthermore, it also contains various protective proteins, which exert antibacterial (63) and immunological activities (64).

The potential therapeutic potencies of camel milk on autism might be from the inflammation-inhibiting proteins that help rehabilitating the immune system, or from its antibacterial properties that have effects on certain symptoms accompanying autism enterocolitis (65).

There is one double-blind, placebo-controlled trial to evaluate the effects of camel milk therapy among children with an ASD (64, 66). In that study, 45 subjects were randomly assigned to receive boiled camel milk (*n* = 15), raw camel milk (*n* = 15), and cow milk as placebo (*n* = 15) for 2 weeks. The amount of milk consumption was 500 ml/day. Investigators observed significantly improved clinical measurements (CARS score) recorded by trained professional and parents of autistic children in raw camel milk group only. Camel milk therapy was generally well tolerated with minimal adverse effects (irritability and stomach discomfort).

Although the results appear to be encouraging, the trial has limitations. Sample size was small; mechanisms was not clear; use of cow milk as placebo was not convincing; and only one behavior measure (CARS scores) was inadequate to comprehensively reveal changes in core symptoms of ASD. The study laid a foundation for further detailed researches.

### Gluten and Casein: Free and Supplement

Gluten-free and casein-free (GFCF) diets implies the elimination of all food items containing cereals such as flours and bread, or containing dairy products such as milk, yogurt, butter, and others.

The opioid theory was the most cited theory for adopting of a CFGF diet for the treatment of ASD (67, 68). It is known that, after digestion, certain types of peptides could cross the intestinal mucosa intact and may further cross the blood–brain barrier through bloodstream transportation and reach the central nervous system even to a high level in the case of impairments in intestinal track or celiac disease (69). There is increasing evidence for a “leaky gut” associated with at least some cases of ASD (70, 71). Therefore, digestion of gluten and casein may result in an overload of exogenous neuropeptides, including gluteomorphins and beta-casomorphins (72), which may affect brain functioning as well as produce an opioid-type effect that manifests in the core symptoms commonly observed in ASD.

Six small randomized trials on this topic that has used a blind method have been published. In 2003, Knivsberg et al. published the first randomized, controlled, single-blind study of a GFCF dietary intervention on autistic behavior (73). In their 12 months experimental period on 20 children, significant reduction of autistic behavior, measured by Diagnose of Psykotisk Adfaerd hosBørn, was observed in GFCF diet group, but not in the control group. However, the other two single-blind trials observed no statistically significant differences between treatment groups (74, 75). Of the only two double-blind, placebo-controlled studies, one observed for 12 weeks (76) and one used a 6-week crossover design (77), both data did not support statistically significant effects of GFCF dietary on core symptoms of ASD.

By contrast, Pusponegoro et al. conducted a double-blind, placebo-controlled trial to determine the effect of gluten and casein supplementation on symptoms in children with ASD (78). After 11 g of gluten and 12 g of casein supplementation for 1 week, mean differences in approach withdrawal problem composite score, a subtest of Pervasive Developmental Disorders Behavioral Inventory, and in the gastrointestinal (GI) symptoms were not significant.

Numerous reviews on this topic have been published, and they all concluded GFCF diet as lack of evidence for improving autistic symptoms (67, 68, 79–81). Overall, findings from current best evidence suggest that GFCF dietary may not help to reduce core symptoms of ASD neither gluten nor casein supplementation increase the symptoms. As GFCF dietary are a special version of common food items that are usually expensive (82), administration of such a diet may place an extra burden on families. Besides, long term following GFCF diet will significantly change the children’s eating behavior and can further complicate the social integration of children with ASD (83, 84). Thus, for the treatment of core symptoms of ASD, the GFCF diet is not recommended.

### Omega-3 Fatty Acids

Omega-3 fatty acids are a special group of long-chain polyunsaturated fatty acids that play an important role in normal growth and are essential to neurodevelopment (85–87). The two omega-3 acids that are of interest for supplementation are eicosapentaenoic acid (EPA) and docosahexaenoic acid (DHA) as human body cannot synthesize them and the intake of EPA and DHA are mostly depend on dietary or food supplements (88).

Eicosapentaenoic acid and DHA are substrate for the production of eicosanoids such as prostaglandins (89) which is necessary for cellular communication (90) and immune regulation (91). EPA and DHA are of fundamental importance for brain structure and function because they are orthomolecules and their functional sites are exclusively cell membranes (92). Therefore, DHA and EPA have been studied for the treatment of multiple neurodevelopmental disorders, including ASD (93, 94), ADHD (95, 96), schizophrenia (97), and mood disorders (98, 99). Also, reduced concentrations of omega-3 fatty acids have been observed in children with ASD (10, 100), suggesting omega-3 fatty acids supplementation may be of help for the treatment of symptoms associated with ASD.

There have been six double-blind, placebo-controlled trials, with inconsistent results. Amminger et al. (101) observed 840 mg/day EPA + 700 mg/day DHA for 6 weeks compared with placebo was superior over placebo in reducing stereotypy, inappropriate speech and hyperactivity, though their statistical analyses for ABC subscales indicated no significant differences between treatment groups. In 2015, Mankad et al. (102) published a replication study with the same dose as Amminger et al. and longer duration (12 weeks), but failed to any effect of the supplementation on core symptoms of ASD. In a pilot trial, Bent et al. (103) used 0.7 g/day EPA + 0.46 g/day DHA for 12 weeks for the treatment of ASD in 27 children aged 3–8 years, their initial results did not show a statistically significant benefit from omega-3 fatty acids. Several years later, Bent et al. (104) published their new findings from a trial with larger sample size (*n* = 57), they observed a significant improvement in stereotypy and lethargy subscales of ABC, but no significant differences in other measures such as SRS and CGI-I. In another pilot study, Yui et al. (105) used a 12 weeks, 0.24 g/day DHA + 0.24 g/day arachidonic acid (ARA) intervention on 13 older ASD patient (age range: 6–28 years). Significant improvements were observed in social withdrawal subscale of ABC, stereotyped and repetitive behavior of Autism Diagnostic Interview-Revised, and communication subscale of SRS, this was the only study that reported significant improvements in all behavior measures. Voigt et al. (106) measured the effect of long-term (6 months) intake of low dose DHA (200 mg/day) on 48 children with ASD, and they did not find a significant difference in CGI-I or in other measures across groups.

Among the six included RCTs, five showed that omega-3 fatty acid supplementation does not affect core symptoms of ASD in overall measures, and the only study that reported the most significant results was clearly limited in sample size (105). Besides, a recent meta-analysis of five RCTs have assessed the association, and results from their quantitative analyses indicated that there were no statistically significant differences in ASD symptoms between groups measured by validated scales (93).

Currently, limited data suggest that omega-3 fatty acid supplementation may be not effective for the treatment of core symptoms of ASD in children. However, fatty acids were generally safe and were well tolerated in all six trials, and they might still be of benefit for the neurodevelopment in children.

### Probiotics and Digestive Enzyme

Probiotics are microorganisms that are living in intestinal mucosa and are able to increase expression of mucin (107), reduce over growth of bacteria (108), stimulate mucosal immunity (109, 110), and synthesize antioxidant substances (111), and thus stabilize the mucosal barrier and improve digestive health (112, 113).

Studies reported a high prevalence of various GI disturbances in patients with ASD (114, 115), and there is increasing evidence for a “gut–brain axis” associated with at least some cases of ASD (116, 117). Those suggest that the development of treatment strategies that can restore normal gut microbiota, reduce gut production and absorption of toxins, such as dietary supplement of probiotics, may provide an optional strategy that may attenuate behavioral symptoms of ASD by easing GI symptoms.

A double-blind placebo-controlled trial using a crossover design over 12 months on 22 children aged 4–16 years with ASD showed significant improvement in Total Behavior Problem Score, but not in Development Behavior Checklist with daily feeding of 4.5 × 10^10^ CFU of probiotics (118). While another double-blind placebo-controlled trial (119) using crossover design over 6 months for 43 children aged 3–8 years did not find any clinically significant improvement in all behavioral measures with the Peptizyde™, a combination of three plant-derived proteolytic enzymes. Saad et al. performed a double-blind RCT on 101 children with ASD aged from 3 to 9 years. Significant improvement in emotional response, general impression autistic score, general behavior, and GI symptoms was observed after 3 months digestive enzyme therapy (120). Samples in those studies were relatively small but not limited to children with GI symptoms. Although probiotics and digestive enzyme treatment of core symptoms of ASD were still inconclusive, they at least did improve the GI health in ASD patients.

Currently, a new RCT using a rigorous control design aimed to determine the role of probiotics on clinical, biochemical, and neurophysiological parameters in ASD children is underway (121). The method protocol used a group of 100 preschoolers with ASD and classified to a GI group or to a non-GI. Findings from this trial could add more information on the effects of treatments with probiotics on children with ASD.

### Sulforaphane (SFN)

Sulforaphane, an isothiocyanate derived from cruciferous vegetables, is the product of glucoraphanin hydrolysis by myrosinase (122, 123). Although the mechanism remains to be clarified, the “fever effect” of ASD that can dramatically but temporarily ameliorate the behavioral symptoms of ASD was reported by parents of children with ASD (124). SFN has metabolic effects that extensively associated with heat shock proteins (125), and thus in some ways resemble that of fever (125). Also, several biological effects of SFN such as against oxidative stress (126), inflammation (127, 128), and DNA damage (129), which are potential and prominent mechanistic characteristics of ASD, and its low toxicity (130) and well tolerance premised the test of SFN treatment of ASD.

Singh et al. (131) conducted and reported the first double-blind, placebo-controlled trial of SFN treatment of ASD on 29 males aged 13–17 years old for 18 weeks and observed significant improvement in all behavioral measures (ABC, SRS, and CGI-I), especially in social interaction and communication. It appears that SFN is safe and effective and might be acceptable for daily supplement. However, participants in this trial were older and were selected because of parental reported history of reduced ASD symptoms during febrile episodes, and participants received an uncharacteristically low placebo effect (<3.3%).

A recent review outlined several other ASD-associated basic physiological pathways that can be regulated by SFN, including redox metabolism/oxidative stress, mitochondrial dysfunction, immune dysregulation/neuroinflammation, febrile illness and the heat shock response, and synaptic dysfunction (132). Those pathways may guide further novel treatment strategies for the improvement of core and associated symptoms of ASD. Currently, there are five ongoing trials that are further investigating SFN treatment of ASD (NCT02654743, NCT02909959, NCT02677051, NCT02561481, and NCT02879110). New findings on safety and treatment effect of SFN are gradually to be reported.

## Discussion

While the exact etiology of ASD remains unknown, epidemiological studies have evidenced various risk factors, though none has been proved to be necessary or sufficient alone for developing ASD (133). Large population-based investigations provided novel insights into environmental risk factors for ASD and twin studies served as a unique platform for studying the genetics (134). The gene–environment interplay is dynamic in autism, and our understanding is still at an early stage. No medication has been shown to reliably improve core symptoms of ASD; however, dietary supplements intervention as a kind of complementary and alternative therapy is widely used. Although, in most cases, dietary supplements are used for the symptoms in children with ASD, and only have a minor role in the treatment of ASD so far, they are potentially effective and might be of help for elucidating the pathogenesis.

Among the eight different supplements reviewed, three (methyl B12, vitamin D3, and omega-3 fatty acids) were guided by deficiencies in ASD; three (folinic acids, probiotics, and CFGF diet) were guided by potential etiological theory and two (camel milk and SFN) were guided by anecdotal evidence. Summarized number of evidence and recommendations were presented in Table 2.

**Table 2:** Summary of evidence and recommendations.

Supplement	Number of evidence	Rationale/mechanism	Recommendation
Methyl B12	2 double-blind RCTs	Correct deficiency	Effectiveness inconclusive but acceptable
Vitamin D	1 double-blind RCT	Correct deficiency	Promising
Omega-3 fatty acids	6 double-blind RCTs	Correct deficiency	Less effective but acceptable
Probiotic and digestive enzyme	3 double-blind RCTs	Ease GI symptoms	Promising
Folinic acid	1 double-blind RCT	Correct deficiency	Promising
Camel milk	1 double-blind RCT	Improve immune function	Promising
		Ease GI symptoms	
Sulforaphane	1 double-blind RCT	Fever effect	Promising
Gluten-free and casein-free diet	5 double-blind RCTs	Decrease exogenous neuropeptides	Not recommend

*GI, gastrointestinal; RCT, randomized controlled trial*.

An ideal dietary supplement for children should be safe, easy, cheap, and sensible (135). For safety, all eight different supplementations were generally safe and well tolerated with no report of serious adverse event; for easiness, all eight supplements were easily available and seven could be easily orally administrated, only methyl B12 were injected in current trials and its effect by oral delivery should be further tested; all supplements except GFCF diet were reasonable in price and might be acceptable for families; as for the treatment effect, GFCF diet and ometa-3 fatty acid were concluded as ineffective on core symptoms of ASD; while vitamin D is promising and may be recommended for children with ASD; effects of the remaining supplements were inconclusive based on current evidence and further investigations were needed, but folinic acid and SFN were most promising.

It should be noted that the therapeutic effect of some supplements may be only suitable for children with specific conditions. For example, SFN might be more efficacious in children with parental reported history of “fever effect,” and the improvements in autistic symptoms after folinic acid supplementation was significantly greater in FRAA-positive patients. Also, it is important to mention that although several dietary supplements may not have benefit in improving core symptoms of ASD, they still could be considered for complementary and alternative treatments for reversing other symptoms such as probiotics for GI symptoms and omega-3 fatty acids for low plasma fatty acid levels in autistic children. Careful examinations should be performed before using dietary supplements as a complementary therapy.

There are several limitations in our review process. First, the number of included studies was limited. For instance, according to the inclusion criteria, we excluded open-label trials of vitamin D supplementation in case of placebo effect, thus leaving only one RCT included, but there were two open-label trials (136, 137). Second, we were unable to perform quantitative analysis of current evidence due to significant heterogeneity in interventions and in outcome measures, thus we only reviewed studies qualitatively. Third, the outcome of interest was limited to core symptoms of ASD, several other supplements used for other aspects of ASD was not considered, such as melatonin, which was used to help autistic children with sleep problems (138).

### Future Directions

- The identification and validation of the cellular targets and mechanisms of supplements are keys for contributing to understanding of their health benefits as well as etiology of ASD.
- Further studies on varied dosages of supplements that are of potential benefits are warranted to maximizing the benefits and minimizing the risk.
- Study durations should be prolonged and sample size should be enlarged as dietary supplements are generally long-time administrated.
- Reports of anecdotal evidence of treatment effects of novel supplements on symptoms of ASD are encouraged.

## Conclusion

To date, most RCTs were limited in small sample sizes and were conducted with various populations and study groups. Many discrepancies and conflicting information in patients must be resolved before recommending a supplement as a safe and effective alternative approach for the treatment of ASD. Clearly, additional clinical trials should be conducted. We believe that a joint effort among basic and clinical researchers and clinicians is paramount to further advance our understanding of the treatment potentials of dietary supplements as well as discovering of novel potent therapeutics.

## Author Contributions

Y-JL and D-XX defined the focus of the review. Y-JL and J-JO searched and screened the papers for inclusion. Y-ML summarized included papers. Y-JL drafted the manuscript. All the authors contributed to reviewing the manuscript and read and approved the submitted version.

## Conflict of Interest Statement

The authors declare that the research was conducted in the absence of any commercial or financial relationships that could be construed as a potential conflict of interest.
